# Comparison of Dosimetric Parameters in Dysphagia Aspiration-Related Structures and Clinical Correlation in Head and Neck Cancer Patients Treated With Radiotherapy

**DOI:** 10.7759/cureus.26765

**Published:** 2022-07-11

**Authors:** Prachi Upadhyay, Piyush Kumar, Arvind Kumar Chauhan, Pavan Kumar, Jitendra Nigam, Navitha S

**Affiliations:** 1 Radiation Oncology, Shri Ram Murti Smarak (SRMS) Institute of Medical Sciences, Bareilly, IND

**Keywords:** ryle's tube, 3d-crt, head and neck cancer, imrt, radiation oncology, dars

## Abstract

Introduction

Chemoradiotherapy plays a major role in the treatment of head and neck cancer (HNC). Persistent dysphagia following primary chemoradiotherapy for head and neck cancers can have a devastating effect on a patient’s quality of life. Many studies have shown that the dosimetric sparing of critical structures which were included in swallowing such as the pharyngeal constrictor muscle and larynx can provide improved functional outcomes and better quality of life. However, there are no current randomized studies confirming the benefits of such swallowing-sparing strategies. The aim is to evaluate late dysphagia after chemoradiotherapy for head and neck cancer and to examine its correlation with clinical and dosimetric parameters.

Materials and methods

The period of this prospective study was from November 2018 to March 2020. Patients were divided randomly in 1:1 ratio into two groups, group 1 and group 2, each with 25 patients. Group 1 was planned by three-dimensional conformal radiotherapy (3D-CRT) technique and group 2 was planned by intensity-modulated radiotherapy technique (IMRT) technique. Treatment was delivered after approval of radiotherapy plan. To evaluate the dose to dysphagia aspiration-related structures (DARS), these structures were contoured and dose-volume histograms were generated. Various dosimetric parameters of DARS were evaluated. Swallowing status was clinically evaluated based on the Radiation Therapy Oncology Group and the Common Terminology Criteria for Adverse Events, version 5.

Results

A significant advantage was seen with intensity-modulated radiotherapy technique (IMRT) in comparison to three-dimensional conformal radiotherapy (3D-CRT) in terms of mean dose delivered to the pharyngeal constrictor muscles (66.03 Gy vs 68.77 Gy, p=0.003). The mean dose delivered to the combined dysphagia/aspiration-related structures (DARS) was statistically significantly lower in IMRT compared to 3D-CRT (66.15 Gy vs. 70.09 Gy, p<0.001). Other dose-volumes were also reduced in IMRT group (V30: {98.64% vs. 99.88%, p=0.05}; V50: {90.49% vs. 99.02%, p=0.0002}; V60: {83.92% vs. 95.04, p=0.0002}; D50: {70 Gy vs. 71.16 Gy, p=0.001); and D80: {61.18 Gy vs. 67.39 Gy, p=0.01}. Futhermore, the clinical worsening of dysphagia was less common in IMRT group (48% vs. 80%, p=0.039).

Conclusion

IMRT can reduce the high-dose volumes received by the DARS receiving high doses by sparing these structures through optimization. This may provide a significant additional benefit that could improve dysphagia and hence the quality of life of patients with head and neck cancer.

## Introduction

A multidisciplinary approach is necessary for the management of head and neck cancer. Treatment options include surgery, radiotherapy, chemotherapy, and a combination of these modalities. Chemoradiotherapy is the main treatment used for patients with locally advanced head and neck cancer (HNC) who are not suitable to undergo surgical treatment, or when tumor is not operable. Treatment with definitive radiotherapy or chemoradiotherapy is generally associated with complex-shaped, large target volumes surrounded by various critical structures (e.g., the spinal cord, salivary glands, optic structures, and structures involved in swallowing). Radiotherapy-induced dysphagia represents a substantial burden in this context, with nearly 50% of patients highlighting it as a distressing symptom one year after treatment completion [1]. It has a devastating impact on a patient’s physical, social, and emotional state. A major clinical consequence of dysphagia is aspiration and related pneumonia. This is typically under-reported in most HNC trials, as assessments are only undertaken at the onset of clinical symptoms, thereby failing to detect silent aspiration.

The intensity-modulated radiotherapy (IMRT) in HNC reduces the dose delivery to parotid glands and has improved quality of life by reducing incidence of xerostomia. It can also reduce dose to critical swallowing- structures, such as the pharyngeal constrictor muscle and supraglottic larynx. This realization has shifted the focus of treatment towards identifying organs at risk and evaluating the potential of dose constraints.

However, to date, no randomized controlled trials have been conducted to confirm the benefits of such swallowing-sparing strategies. Furthermore, in developing countries such as India, three-dimensional conformal radiotherapy (3D-CRT) remains a more accessible and affordable option for the majority of patients with HNC.

In the present study, we assessed the role of late dysphagia after chemoradiotherapy (IMRT and 3D-CRT) for HNC and examined the association of these treatment options with clinical and dosimetric parameters.

## Materials and methods

This study is approved by the Institutional Review Board of Shri Ram Murti Smarak Institute of Medical Sciences under the approval number SRMSIMS/ECC/2018-19/214. A total of 50 patients with HNC who had received chemoradiotherapy in our institution from October 2018 to March 2020 were selected for inclusion in this study. None of the patients had received any other modality of treatment as primary (surgery or chemotherapy), and none had distant metastasis. Patients were randomly assigned in 1:1 ratio into two groups (25 patients per group). In group 1, the 3D-CRT five-field approach was employed, and in group 2, IMRT was used.

Radiotherapy planning and technique

Immobilization and Simulation

Patients were immobilized on a base plate in the extended-neck position using a fixed five-point thermoplastic cast, with an individualized supportive neck rest. A radiotherapy-planning contrast-enhanced computed tomography (CECT) scan with 3 mm slice thickness was obtained in the supine position with three radiopaque fiducial markers.

Delineation of Structures

Delineation was performed as follows: (1) primary gross tumor volume (GTV) and clinical target volume (CTV) included the entire primary sub-site; (2) nodal volumes were defined as the draining nodal region related to the primary volume. Department protocol follows the guidelines for the delineation of neck node levels for head and neck tumors as described by Gregoire et al. [2]; (3) CTV final included CTV primary and nodal, and planning target volume (PTV) is institutional, i.e., 7 mm.

Delineation of Organ at Risk Structures (OARs)

The delineated OARs were the left and right parotid glands, spinal cord, brain stem, eyes, lens, optic chiasma, optic nerve, left and right cochlea, lips, and mandible. They were delineated as per the Danish Head and Neck Cancer Study Group (DAHANCA) guidelines [3]. An isotropic expansion of 5 mm was used for planning OAR volume (PVR) of the spine and 3 mm for that of the brainstem.

Dysphagia/Aspiration-Related Structures (DARS)

The swallowing structures or “dysphagia/aspiration‐related structures” were identified as suggested by Christianen et al. [4]. They included - superior constrictor muscles (SCM), middle constrictor muscles (MCM), inferior constrictor muscles (ICM), base of tongue (BOT), larynx, cricopharyngeus muscle/upper esophageal sphincter (UES). A combined volume for all the swallowing structures was also generated, (termed DARS), and different doses in relation to the volumes were determined.

Planning: 3D-CRT Technique (Five Field Technique)

Three-dimensional CRT technique is a completely isocentric technique with the following five photon beams: two lateral anterior and two posterior oblique fields with gantry an angle of 85, 275, 225, and 135 degrees, respectively (varying by approximately 5-15 degrees), and a posterior field of 180 degrees were used. The fields were partially opened and the spinal PRV was shielded.

In the first phase of treatment, a total dose of 50 Gy was delivered. In the second phase, the off-cord technique was used and the remaining dose of 20 Gy was delivered by two bilateral opposing fields. The dose to the PTV overlying the spinal cord was compromised after a cumulative dose of 50 Gy to the spinal PRV.

IMRT Planning

Coplanar 7-9 fields around the isocentre using isotropic gantry angles were used and adjusted slightly to avoid beam entry through the OARs. In the subsequent step of fluence optimization, the minimum and maximum dose coverage required for the PTV and the dose tolerance of the OARs were defined.

Prescription

The total prescribed dose to the planning target volume was 70 Gy in 35 fractions over seven weeks in five days per week, along with weekly concurrent chemotherapy, i.e., injection cisplatin 35 mg/mt^2^ I/V in both groups.

Assessment of toxicity

Radiation toxicity (skin, mucosal, laryngeal, salivary gland, and hematologic toxicity) was assessed by using Radiation Therapy Oncology Group (RTOG) acute and late morbidity scoring criteria. During chemoradiation, patients were assessed weekly for acute radiation reactions. The acute RTOG morbidity scoring criteria were applicable from the day of commencement of radiotherapy until 90 days thereafter. Assessment for late radiation reactions was performed using the RTOG late morbidity scoring criteria, applicable from 90 days onward.

During treatment, adverse event assessments performed on a weekly basis, and thereafter monthly, using the Common Terminology Criteria for Adverse Events, version 5.0 (CTCAE). Patients were assessed for dysphagia weekly during radiotherapy, after radiotherapy ceased, and monthly thereafter for six months. Detailed subjective grading was used as per the CTCAE. Grade 1 - symptomatic, able to eat a regular diet; grade 2 - symptomatic, altered eating/swallowing; grade 3 - severely altered eating/swallowing, tube feeding, total parenteral nutrition; grade 4 - life-threatening consequences, urgent intervention indicated.

Plan evaluation

Dosimetric parameters of PTV (V95%, V90%, Dmax, Dmean, conformity index, and homogeneity index) along with the mean dose for individual swallowing structures, as well as for combined swallowing structures, were tabulated for the 3D-CRT and IMRT groups. Moreover, V30, V50, V60, V70, D50, D80, and Dmean for the constrictor muscles (superior, middle, and inferior), base of the tongue, larynx, UES, and combined DARS were evaluated and documented. No initial dose constraints were prescribed for planning purposes. The observations of each patient (clinical and dosimetric) were tabulated or graphically represented.

Statistical analysis

Patients were analyzed on intention-to-treat basis. Statistical significance was calculated using an unpaired t-test of unequal variances, and chi-square test was used for the analysis of compliance and toxicities. A p<0.05 was considered statistically significant.

## Results

Among the sample population, the majority of the patients had oropharyngeal cancer; 91% in the 3D-CRT group and 50% in the IMRT group. In patients with oral cavity and laryngeal carcinoma, the incidence of post-treatment dysphagia was higher in the 3D-CRT than in the IMRT group; however, statistical significance could not be determined owing to the small sample size. In patients with oropharyngeal and hypopharyngeal carcinoma, this incidence was also higher in the 3D-CRT than in the IMRT group; however, the difference was not significant (Table 1). The incidence of dysphagia was higher in the 3D-CRT than in the IMRT group (80% in the 3D-CRT group and 48% in the IMRT group) (Table 2).

**Table 1:** Incidence of post-treatment dysphagia by cancer site. IMRT: intensity-modulated radiotherapy, 3D-CRT: three-dimensional conformal radiotherapy

Site	IMRT% (n)	3D-CRT% (n)	p-Value
Oral cavity	0% (0/1)	80% (4/5)	-
Oropharynx	50% (8/16)	91.6% (11/12)	0.1
Hypopharynx	60% (3/5)	60% (3/5)	0.5
Larynx	33% (1/5)	66.6% (2/3)	1

**Table 2:** Clinical post-treatment dysphagia pattern for both techniques. IMRT: intensity-modulated radiotherapy, 3D-CRT: three-dimensional conformal radiotherapy

Technique	Dysphagia	No dysphagia	p-Value
3D-CRT	20	5	0.039
IMRT	12	13	

None of the patients receiving either treatment techniques achieved the recommended dose constraints of Dmean <50 Gy as mentioned in the Quantitative Analyses of Normal Tissue Effects in the Clinic (QUANTEC). Nevertheless, dosimetry analysis of the mean dose delivered to the pharyngeal constrictor muscles for both techniques found a statistically significant difference (p=0.03) (Table 3). Corresponding with this, the worsening of dysphagia was significantly less in the IMRT than that in the 3D-CRT group (12 patients {48%} vs. 20 patients {80%} p=0.039) (Table 2).

**Table 3:** Mean dose to pharyngeal constrictor muscles (SCM, MCM, ICM). IMRT: intensity-modulated radiotherapy, 3DCRT: three-dimensional conformal radiotherapy, SCM: superior constrictor muscles, MCM: middle constrictor muscles, ICM: inferior constrictor muscles

Variable	3D-CRT	IMRT	p-Value
Mean dose	68.77 Gy	66.03 Gy	0.03

Worsening of post-treatment dysphagia was more commonly experienced in the 3D-CRT group than that the IMRT group. As for the combined DARS, the association between the mean dose of 66.15 Gy (Table 4) and the worsening of dysphagia showed a trend towards significance (p=0.07) (Table 5). However, no significant difference can be reported, as no constraints were prescribed.

**Table 4:** Mean dose to the dysphagia/aspiration related-structures. IMRT: intensity-modulated radiotherapy, 3D-CRT: three-dimensional conformal radiotherapy, DARS: dysphagia aspiration-related structures

Variable	3D-CRT	IMRT	p-Value
Mean dose	70.09 Gy	66.15 Gy	<0.0001

**Table 5:** Comparison of the mean dose to dysphagia/aspiration-related structures and the incidence of dysphagia in the IMRT group. IMRT: intensity-modulated radiotherapy

Mean	Dysphagia	No dysphagia	p-Value
>66.15 Gy	9	4	0.07
<66.15 Gy	3	9	

Ryle’s tube dependence was more common in the 3D-CRT group (n=8, 3.6%) than the IMRT group (n=6, 28%); however, the difference was not significant (p=0.52). Some patients were advised about tube dependence although, owing to discomfort, they refused (Table 6).

**Table 6:** Comparison of Ryle's tube dependence during treatment. IMRT: intensity-modulated radiotherapy, 3D-CRT: three-dimensional conformal radiotherapy

Group	Need for Ryle’s tube	No need for Ryle’s tube	p-Value
3D-CRT	9 (36%)	16 (64%)	0.52
IMRT	7 (28%)	18 (72%)	

## Discussion

In this study comparing the incidence of dysphagia after 3D-CRT and IMRT, we found that IMRT is potentially clinically important, as it has a significant dose-volume relationship with dysphagia and aspiration, which can serve as an initial dosimetric goal for IMRT. This finding supports the hypothesis that reducing the radiation dose to the DARS may reduce the incidence and severity of dysphagia. Clear dose constraints have been previously defined to decrease the incidence and severity of xerostomia. Unfortunately, the same guidelines have not yet been established for dysphagia. Deantonio et al. and Feng et al. reported late dysphagia (all grades) rates of 60% and 56%, respectively; our study reported a similar rate at 64% [5,6].

To reduce the incidence of dysphagia after IMRT, it is important to identify and delineate the DARS. Eisbruch et al. were the first to report that radiation damage to pharyngeal constrictors and larynx was implicated in post-radiation dysphagia [7]. They had suggested that reducing the radiation dose to these structures may lead to improved swallowing outcomes. Following this, many trials have been initiated to establish whether dose reduction to the DARS can improve swallowing outcomes in HNC treated with IMRT. The results of these studies consistently show that an increased radiation dose to larger volume of pharyngeal constrictors results in worse dysphagia.

Relevant doses to individual structures involved in swallowing and aspiration have been suggested by several studies. Levendag et al. reported a 19% increase in the probability of dysphagia with every additional 10 Gy delivered to the SCM and MCM [8]. Li et al. suggested that, in order to reduce the risk of prolonged gastrostomy feeding tube use, the dose to the ICM should be constrained to a mean of <55 Gy with a maximum dose of <60 Gy to the UES [9]. As stated in QUANTEC, the mean dose constraint to constrictor muscles is <50 Gy; however, in our study, none of the patients undergoing either of the radiation techniques received a dose that complied with this limit. Nonetheless, in terms of pharyngeal constrictor muscles, a significant difference between 3D-CRT and IMRT was found in the dosimetry analysis (p=0.03). Correspondingly, the worsening of dysphagia was significantly lower in the IMRT group compared to the 3D-CRT group (12 patients {48%} vs. 20 patients {80%}; p=0.039). This highlights that improved dose delivery to the constrictor muscles translates into a clinically meaningful and significant reduction in the incidence of dysphagia.

A study by Prameela et al. compared radiation dose to the DARS in IMRT and 3D-CRT, and its relationship with swallowing function [10]. For IMRT and 3D-CRT, the mean dose for the SCM, MCM, and ICM was 62.56 Gy vs. 57.44 Gy; 49.54 Gy vs. 55.75 Gy; and 42.17 Gy vs. 53.75 Gy, respectively (p=0.65, p=0.09, and p=0.023, respectively). The degree of dysphagia was comparatively reduced after IMRT; the reduction proved to be significant in the case of the ICM muscles. Similar to the findings of Prameela et al., in the present study - for IMRT compared to 3D-CRT - we found a non-significant reduction in the degree of dysphagia after the mean dose delivered to the SCM and MCM (66.18 Gy vs. 69.02 Gy; 70.27 Gy vs. 69.68 Gy; p=0.11, p=0.26); however, contrary to their findings, the reduction after the mean dose delivered to the ICM was not significant (66.35 Gy vs. 67.33 Gy; p=0.50) [10]. The difference in findings may be explained by the total prescribed dose in some of the plans used in the study by Prameela et al. being 66 Gy, whereas, in our study, a uniform dose of 70 Gy was prescribed to all patients [10].

In a study among 55 patients, van der Molen et al. estimated the dose-volume parameters for swallowing (SCM, MCM, and ICM) and mastication structures (e.g., the masseter) treated with IMRT and its relationship with swallowing problems [11]. They reported mean dose constraints to SCM, ICM, and MCM of 63.0±10.0 Gy, 63.3±11.4 Gy, and 56.9±14.4 Gy, respectively. Our study showed the dose delivered to the constrictor muscles to be nearly comparable to the constraints defined in their study. Another study, conducted by Turkkan et al. among 26 patients treated with 3D-CRT, evaluated the constraint relationship of late dysphagia with dosimetric and clinical parameters in patients receiving radiotherapy for head and neck cancer [12]. They reported mean dose constraints to the SCM, MCM, and ICM of 58.03±10.73 Gy, 64.16±3.89 Gy, and 64.13±5.13 Gy, respectively (p≤0.0001 for the SCM, p=0.036 for the ICM). The delivered dose to the constrictor muscles was not in compliance with QUANTEC recommendations of a Dmean of 50 Gy. This was due to large volume of the DARS located outside the PTV being exposed to a high dose in the 3D-CRT plans.

In a study among 92 patients each receiving 3D-CRT and IMRT, Al-mamgani et al. compared the toxicity of both techniques along with a boost provided using brachytherapy; they reported a reduced mean dose to SCM, MCM, and ICM when using IMRT vs. 3D-CRT (56.5 Gy vs. 69.9 Gy, 52.4 Gy vs. 63.4 Gy, and 41.5 Gy vs. 52.2 Gy, respectively) [13]. Similar results were found in the present study, with the reduced mean dose to SCM, MCM, and ICM when using IMRT in comparison to 3D-CRT (66.18 Gy vs. 69.02 Gy; 70.27 Gy vs. 69.68 Gy; and 66.35 Gy vs. 67.33 Gy (p=0.11, p=0.26, and p=0.50), respectively. Additionally, we found a significant reduction in high-dose volume (V50: 91.1% vs. 99.62%, p=0.04 for the SCM; 99.93% vs. 100% p=0.30 for the MCM; 91.79% vs. 99.93%, p=0.009 for the ICM). The improved dose delivery to the constrictor muscles in the IMRT plans is due to improved sparing of the volume of constrictor muscles located outside the PTV. However, in the 3D-CRT plans, the treated volumes were larger than the PTV. This possibly explains that on retrospective evaluation of dose delivery to the DARS, it was tedious to restrict the dose in 3D-CRT aimed at the constrictor muscles. The QUANTEC dose constraint of 50 Gy could not be achieved by either method; nevertheless, IMRT allowed for a significant dose reduction over 3D-CRT. In our study, beam optimization was not utilized to spare the DARS; it might have allowed for improved sparing and achieving the constraints.

The following question that arises is the incidence of late dysphagia in relation with tumor location. Both Caudell et al. and Dirix et al. found that primary tumor sites in the larynx, hypopharynx, and posterior pharyngeal wall were associated with long-term dysphagia [14,15]. The incidence of late grade 2-3 dysphagia correlated with the primary tumor location in the oropharynx. This fact can be related to the proximity of the oropharyngeal region to the pharyngeal constrictor muscles, in particular, the SCM and the MCM, that consequently received the same dose. Similar results were found in our study; post-treatment dysphagia occurred most often in oropharyngeal followed by hypopharyngeal cancer.

The study by Prameela et al. compared radiation dose delivered to the DARS in IMRT and 3D-CRT, and its relation to post-treatment swallowing status, in patients with carcinoma of the anterior two-thirds of tongue [10]. In IMRT, the mean V30 dose received by the DARS was significantly less compared than that of 3D-CRT (96.97 Gy vs. 95.68 Gy, p=0.051). Furthermore, there was a significant reduction in high-dose volume parameters in IMRT (V50: p=0.002), (V60: p=0.002), and (D80: p=0.023). Similar findings were observed in our study, with IMRT showing a significant advantage over 3D-CRT in sparing the combined DARS. The mean V30 dose was significantly reduced (98.64 Gy vs 99.88 Gy, p=0.05), along with high-dose volume parameters (V50: 90.49 vs. 99.02, p=0.0002 and V60: 83.92 vs. 95.04, p=0.0002). Compared to 3D-CRT, significant advantage was also seen with IMRT in terms of D50, D80, and Dmean (70 Gy vs. 71.16 Gy, p≤0.0001; 61.8 Gy vs. 67.39 Gy, p=0.01; and 66.15 Gy vs. 70.09 Gy, p=0.001).

A large volume of the DARS was located within the PTV, which posed a major challenge in its sparing. For radiation oncologists, decreasing the PTV margins to spare DARS will not be a wise decision because it may lead to failure of treatment in terms of local disease control. Incorporating DARS dosimetry in beam optimization may allow for more efficient sparing and a possible reduction in dysphagia. This will possibly avoid treatment gaps and decreased dependency on Ryle's tube, which will possibly improve the quality of life of patients with head and neck cancer. In the literature, no dose constraints have been defined for the combined DARS, only individual structure constraint studies have been conducted. Our study determined a cumulative DARS dose of 66.5 Gy, which requires further analysis.

## Conclusions

This study demonstrated that IMRT is feasible when aiming to spare the swallowing structures. Significant relationships were found between dose-volume parameters in these structures especially base of tongue and larynx, correlating with the objective and subjective measures of swallowing dysfunction and dysphagia. Consequently, these relationships can serve to define optimization goals and motivate efforts to reduce these doses as much as possible. A longer follow-up time is required and more studies are needed to define dose constraints. Most importantly, care in outlining targets in the vicinity of these structures, avoiding target under-dosing, and determining and reporting the locations of loco regional recurrences are essential to ensure that the rates of local recurrences do not increase after IMRT.
